# Parry–Romberg Syndrome: Radioclinical Dissociation in a Paucisymptomatic Form and a Proposed Diagnostic Framework

**DOI:** 10.3390/diagnostics16081219

**Published:** 2026-04-19

**Authors:** Cristian Turlea, Andrei I. Cucu, Alexandru Carauleanu, Roxana Covali, Camelia Tamas, Mihnea A. Popa, Victor Constantinescu, Anca P. Morosan, Elena Porumb-Andrese, Iulian Prutianu, Claudia F. Costea, Amelian Bobu, Adriana Hristea, Alexandru Nemtoi

**Affiliations:** 1 Faculty of Medicine, Vasile Goldis Western University of Arad, 310025 Arad, Romania; dr.turlea@gmail.com; 2 Faculty of Medicine and Biological Sciences, Stefan cel Mare University of Suceava, 720229 Suceava, Romania; alexandru.nemtoi@usm.ro; 3 Emerald Medical Center, 700505 Iasi, Romania; 4 Faculty of Medicine, Grigore T. Popa University of Medicine and Pharmacy Iasi, 700115 Iasi, Romania; ale.carauleanu@umfiasi.ro (A.C.); camelia.tamas@umfiasi.ro (C.T.); amihpopa@gmail.com (M.A.P.); victor.constantinescu@umfiasi.ro (V.C.); anca.morosan@umfiasi.ro (A.P.M.); elena.andrese1@umfiasi.ro (E.P.-A.); pruty04@gmail.com (I.P.); claudia.costea@umfiasi.ro (C.F.C.); amelian.bobu@gmail.com (A.B.); 5 Faculty of Medical Bioengineering, Grigore T. Popa University of Medicine and Pharmacy Iasi, 700115 Iasi, Romania; ana.covali@umfiasi.ro; 6 Clinical Emergency Hospital St. Spiridon, 700111 Iasi, Romania; 7 Faculty of Medicine, Carol Davila University of Medicine and Pharmacy, 050474 Bucharest, Romania; adriana.hristea@umfcd.ro; 8 National Institute for Infectious Diseases “Prof. Dr. Matei Bals”, 021105 Bucharest, Romania

**Keywords:** Parry–Romberg syndrome, progressive hemifacial atrophy, en coup de sabre, white matter lesions

## Abstract

**Background and Clinical Significance:** Parry–Romberg syndrome (PRS), also known as progressive hemifacial atrophy, is a rare disorder characterized by progressive unilateral hemifacial atrophy, with potential involvement of the cranial bones and the brain. Although neurological manifestations are frequently described, central nervous system involvement may be subclinical and detectable only through neuroimaging. Owing to its rarity and the lack of standardized diagnostic criteria, most available data derive from case reports and small case series. **Case Presentation:** We report the case of a 38-year-old female patient diagnosed with PRS (stage 2 according to the Guerrerosantos classification), with onset in childhood characterized by left parietal alopecia, followed by progressive left-sided hemifacial atrophy and a linear “en coup de sabre” lesion. Neurological examination was normal, with no history of seizures or focal deficits. Brain MRI revealed ipsilateral cutaneous, subcutaneous, muscular, and osseous atrophy, as well as atrophy of the left parotid and submandibular glands. Additionally, subcortical white matter lesions were identified in the left frontal lobe in the absence of hemispheric cerebral atrophy. **Conclusions:** This case highlights a significant radioclinical dissociation, demonstrating that central nervous system involvement may occur even in clinically stable and paucisymptomatic forms of PRS. This disease may be associated with subclinical intracranial abnormalities, underscoring the need for systematic neuroimaging evaluation even in the absence of neurological manifestations. Based on the available literature and the specific features of the present case, we propose a practical clinical framework and imaging algorithm to facilitate early diagnosis and to contribute to the standardization of the diagnostic approach in this rare disorder.

## 1. Introduction

Parry–Romberg syndrome, also known as progressive hemifacial atrophy, is a rare neurocutaneous disorder characterized by slow and progressive unilateral atrophy of the facial tissues, including the skin, subcutaneous tissue, muscles, cartilage, and, in some cases, the underlying osseous structures and even the cerebral parenchyma [1,2,3,4]. It frequently coexists with the “en coup de sabre” lesion, a specific form of linear scleroderma typically located on the forehead and frontoparietal scalp [2,5,6]. The disease usually affects one side of the face and tends to follow the distribution of one or more branches of the trigeminal nerve, depending on the severity and extent of involvement [1].

The earliest realistic depictions of progressive hemifacial atrophy, later termed Parry–Romberg syndrome (PRS), have been identified in the skulls of mummies and in funerary portraits from Ancient Egypt, suggesting that the disease existed as early as that period [7]. Subsequently, in 1825, the neurologist Caleb Hillier Parry (1755–1822) [8], and later in 1846, Moritz Heinrich Romberg (1795–1873) [9], accurately described the principal clinical features of the syndrome, characteristics that remain valid to this day. The current eponym, Parry–Romberg syndrome, derives from their contributions [10]. In 1871, another neurologist, Albert Eulenburg (1840–1917), proposed a more descriptive term, hemifacial atrophia progressiva (progressive hemifacial atrophy) [11]. Since then, multiple terms have been used interchangeably to refer to PRS, including progressive facial hemiatrophy, progressive hemifacial atrophy, idiopathic hemifacial atrophy, and hemiatrophia faciei progressiva [11,12].

With regard to the etiology of PRS, it remains incompletely elucidated, with multiple pathogenic mechanisms having been proposed. The most frequently cited include autoimmune vascular and inflammatory processes, sympathetic nervous system dysfunction, trauma, as well as genetic and infectious factors [1]. Supporting this theory, cases of PRS have demonstrated perivascular cerebral inflammation, structural degeneration of cerebral and leptomeningeal vessels, hyalinization changes, and partial vascular obliteration, likely resulting from immune-mediated vascular injury and aberrant endothelial regeneration [13,14].

From a clinical perspective, PRS is characteristically manifested by progressive facial asymmetry and may be associated with a wide spectrum of extracutaneous manifestations. The typical onset occurs within the first two decades of life, and the disease course is characterized by an active progressive phase lasting between 2 and 20 years, followed by spontaneous stabilization in the majority of cases [15,16]. The diagnosis of PRS is primarily based on the clinical recognition of progressive hemifacial atrophy, supported by imaging studies that can demonstrate both facial structural changes and associated intracranial abnormalities [16,17].

Given the rarity of this condition and the absence of standardized diagnostic and therapeutic criteria, most data available in the literature derive from case reports and small case series [1]. In this context, the presentation of each individual case contributes to a better understanding of the clinical spectrum and natural history of the disease. As PRS is a rare disorder and robust epidemiological studies in European populations are lacking, its true prevalence cannot be determined with certainty. Some recent reports estimate a prevalence of at least 1 case per 700,000 individuals [18]. To our knowledge, only four cases of PRS have been reported in the Romanian population to date [19,20,21,22].

In this case report, we describe the mild cutaneous and muscular manifestations observed in a 39-year-old female patient with PRS (stage 2 according to the classification proposed by Guerrerosantos et al.) [23]. The patient presented with an abnormal brain MRI, which revealed ipsilateral frontal lobe white matter lesions. Notably, she did not exhibit any significant neurological manifestations of PRS, and her psychomotor and cognitive functions were preserved, features that are typically impaired in cases with central nervous system involvement.

The aim of this report is to present the clinical and imaging particularities of this case, to analyze the diagnostic challenges and therapeutic options in PRS and to propose a practical clinical framework and imaging algorithms aimed at facilitating early diagnosis and promoting a more standardized diagnostic approach.

## 2. Case Report

### 2.1. Clinical Data

We report the case of a 38-year-old Romanian female patient who was diagnosed with PRS at the age of 25. Information regarding birth length, weight, and head circumference was unavailable; however, these parameters were reported as normal. There was no family history of PRS, scleroderma, or other autoimmune disorders. Additionally, there was no history of trauma or infection involving the affected region (left hemiface). The onset of the disease occurred during childhood, initially presenting with alopecia of the left parietal scalp and loss of eyelashes of the left eyelid. Over time, progressive atrophy developed in the left mental region, followed by involvement of the left temporal and frontal areas, leading to the diagnosis of PRS at approximately 25 years of age.

**Figure 1:**
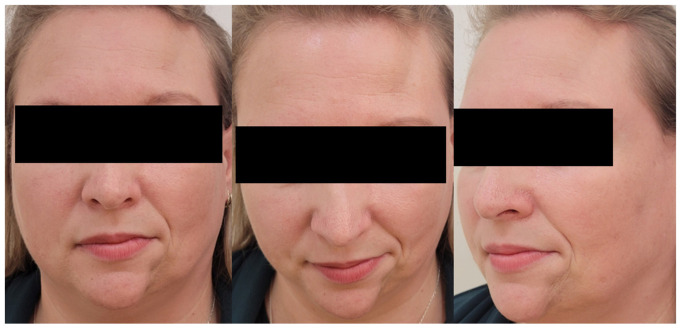
Frontal view, frontal view with anterior head flexion, and left lateral view with slight rotation, demonstrating the characteristic craniofacial asymmetry associated with Parry–Romberg syndrome. Visible findings include a linear frontal “en coup de sabre” lesion, soft-tissue volume loss with atrophy of the left temporalis and masseter muscles, and ipsilateral deviation of the oral commissure and nasolabial fold consistent with unilateral hemifacial atrophy.

Current neurological examination revealed left hemifacial atrophy involving the frontal, left temporal, and left mental regions, as well as a linear frontal lesion consistent with “en coup de sabre” (Figure 1). Inspection also demonstrated deviation of the left oral commissure and left nasolabial fold toward the affected side (Figure 1). Clinical examination further showed hypotrichosis of the left eyelashes and left eyebrow, as well as an apparent mild upper left gingival atrophy in the context of dental implants, without clear pathological significance in relation to the underlying disease. The remainder of the neurological examination was within normal limits, with no sensory or motor deficits, no limb atrophy, normal coordination tests, and a Mini-Mental State Examination (MMSE) score of 30/30. Ophthalmologic and oro-maxillofacial evaluations revealed no pathological findings. Routine laboratory tests were within normal limits. The immunological investigations included antinuclear antibodies, anti-neutrophil cytoplasmic antibodies, extractable nuclear antigens panel, anti-dsDNA antibodies, rheumatoid factor with anti-cyclic citrullinated peptid antibodies, complement factors (C3 and C4), and antiphospholipid antibodies. All results were within normal limits or negative. A skin biopsy was not performed.

### 2.2. Imaging Studies

**Figure 2:**
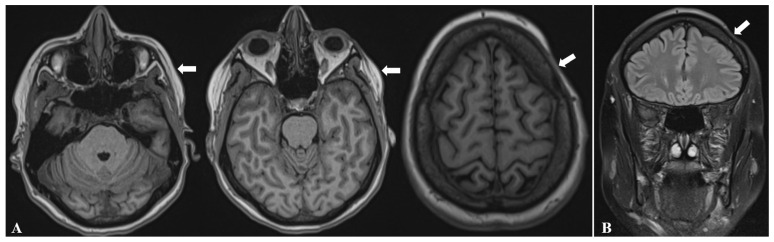
Skin and subcutaneous tissue atrophy involving the left hemiface and the ipsilateral scalp (white arrows), demonstrated on axial T1-weighted (**A**) and coronal T2-weighted brain MRI sequences (**B**).

A head MRI examination, performed for the first time at the age of 38, revealed left-sided hemifacial atrophy involving both the subcutaneous tissue (Figure 2) and the underlying musculature.

**Figure 3:**
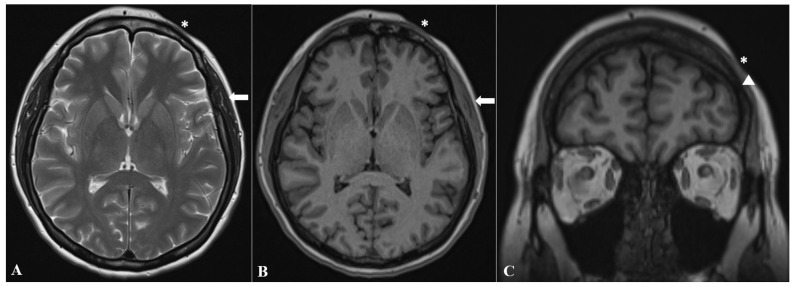
Muscular atrophy (white arrows) and osseous atrophy (white triangle) demonstrated on axial T2-weighted (**A**), axial T1-weighted (**B**), and coronal T1-weighted (**C**) brain MRI sequences. The imaging appearance of the “en coup de sabre” lesion is also visible on these sections, characterized by focal soft-tissue volume loss (white asterisk).

Additionally, brain MRI identified ipsilateral atrophy of the masticatory muscles, specifically the left temporalis and masseter muscles, as well as focal thinning of the left frontal bone (Figure 3). Furthermore, MRI demonstrated soft-tissue volume loss corresponding to the linear morphea lesion at the left frontal scalp (Figure 2B and Figure 3C).

**Figure 4:**
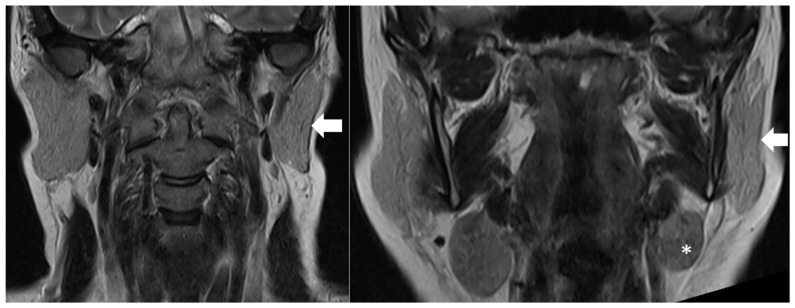
Volume loss of the left parotid gland (white arrow) and left submandibular gland (white asterisk), demonstrated on coronal T2-weighted brain MRI.

Brain MRI further demonstrated ipsilateral atrophy of the parotid and submandibular glands on the side of facial involvement (Figure 4).

**Figure 5:**
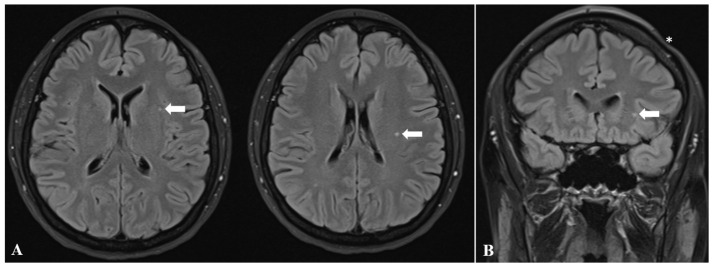
Subcortical white matter lesions in the frontal lobe ipsilateral to the facial atrophy (white arrows), demonstrated on axial FLAIR (**A**) and coronal FLAIR (**B**) brain MRI sequences. Cutaneous atrophy is also visible in subfigure B (white asterisk).

Additionally, FLAIR sequences of the brain MRI demonstrated three subcortical white matter lesions within the left frontal lobe, measuring 4.02 mm, 2.3 mm, and 1.08 mm, respectively (Figure 5). Following administration of the contrast agent, none of the lesions demonstrated enhancement. Overall, contrast-enhanced brain MRI did not reveal any additional abnormalities. Furthermore, time-of-flight (TOF) arterial and venous sequences showed no evidence of cerebrovascular abnormalities.

Following integration of all available imaging findings with the patient’s clinical examination and medical history, a definitive diagnosis of PRS was established, corresponding to stage 2 according to the Guerrerosantos classification [23].

## 3. Discussion

### 3.1. Relevant Epidemiological Data

PRS is a rare disorder with no precisely established incidence [1], as the existing literature is largely limited to case reports and small case series [24]. Some studies estimate an incidence of at least 1 per 700,000 individuals [24,25].

Regarding sex distribution, most studies in the literature report a female predominance [1,2,17,24,26,27], with a female-to-male ratio of approximately 3:1 [1]. The condition typically manifests during the first or second decade of life, with a mean age at onset of approximately 8 years [2]. However, the reported age range is wide, with some authors describing onset between 0.3 and 75 years of age [1,26,28,29,30]. Disease progression generally spans 2 to 20 years before reaching a stabilization phase [8,9,11,31,32,33]. Some authors have reported a mean age at diagnosis of 30.9 years [34], which is comparable to the age at diagnosis of the patient described in the present case report.

The initial diagnosis of PRS is often challenging and may be delayed for several years. It typically requires a comprehensive evaluation, including detailed medical history, thorough clinical examination, imaging studies, exclusion of differential diagnoses, and, in selected cases, histopathological confirmation through biopsy [17]. In this regard, most authors report a median diagnostic delay of approximately 4 years [24,26], with similarly wide ranges from 0 to 41 years [24] and from 0.3 to 45.9 years [26] between disease onset and definitive diagnosis.

The involvement is typically unilateral, affecting the distribution of the trigeminal nerve [2], with atrophy generally confined to one side of the face and not crossing the midline. However, bilateral cases have been rarely reported [26,35,36,37,38], accounting for approximately 2–7.4% of cases according to some authors [24,26]. In such instances, the condition has been classified as Barraquer–Simons syndrome [39], an acquired partial lipodystrophy characterized by progressive and symmetrical loss of adipose tissue.

No clear right- or left-sided predominance has been consistently demonstrated [24]. However, some authors have reported a higher frequency of right-sided involvement (61%) compared to left-sided cases (32%) [34]. In approximately 19–20% of cases, the atrophy extends beyond the face to involve the upper or lower extremities [24,40], most commonly ipsilateral and only rarely contralateral [41]. PRS is frequently associated with linear scleroderma “en coup de sabre,” with various authors reporting coexistence rates ranging from approximately 36% to 53.6%, depending on the studied cohort [24,26,29,42].

### 3.2. Pathogenesis

The etiopathogenesis of PRS remains largely unknown and is considered heterogeneous and multifactorial, with autoimmune, local inflammatory, neurogenic, vascular, infectious, genetic, traumatic, and endocrine factors being implicated. Several mechanisms have been proposed, including hereditary and congenital causes, a history of trauma, abnormalities in fat metabolism, cranial vascular malformations, immune-mediated processes, and dysfunction of the sympathetic nervous system [2,32,43,44,45,46,47]. Among these hypotheses, the autoimmune mechanism currently has the strongest support. Evidence includes the association of PRS with other autoimmune disorders [41], inflammatory changes observed in brain biopsy specimens, and signs of endothelial activation [48]. Additional supporting findings comprise distinct proinflammatory gene expression profiles in skin samples [49], the identification of circulating autoantibodies [50], the presence of oligoclonal bands in cerebrospinal fluid, and the reported improvement of lesions following immunosuppressive therapy [31,32,39,51,52,53,54,55]. Furthermore, some authors suggest that central nervous system (CNS) lesions in PRS represent inflammatory parenchymal processes or may result from immune-mediated vasculitis affecting the CNS [56]. The neurogenic theory currently has limited and largely historical support [41,57], whereas infectious, traumatic, and endocrine factors are considered more likely to act as triggering events in susceptible individuals [58].

Although rare familial cases of PRS have been described [59,60], previous studies have failed to identify a clear pattern of inheritance or a specific causative genetic defect [61,62]. Genetic alterations have been reported in some patients with PRS, including variants in the MTOR and DHX37 genes, as well as neurocristopathies and dysmorphogenetic anomalies [63]. However, these findings have not been conclusively demonstrated to directly determine the clinical phenotype [64]. PRS has also been associated with several congenital conditions, including congenital lower limb hypoplasia [65], congenital ipsilateral cerebral atrophy [66], microphthalmia [39], supernumerary nipple [39], renal malformations [39], congenital torticollis [39], and contralateral Poland syndrome [67]. In addition, PRS has been reported in association with benign tumors such as orbital neurinomas, hamartomas, and mandibular odontogenic fibromas [68]. The coexistence of PRS with migraine and intracranial aneurysms has led to the hypothesis of a neural crest migration disorder, given that many of the tissues affected in PRS, including craniofacial bone and cartilage, smooth muscle, and cranial vasculature, originate from neural crest cells [2,39,69]. Nevertheless, the vast majority of PRS cases are sporadic [1,70,71], and familial aggregations remain rare exceptions in the literature, typically described as isolated case reports [17,60].

With regard to trauma as a potential etiological factor in PRS, studies have reported that between 24% and 34% of patients had a history of preceding trauma [72,73], including surgical trauma (e.g., thyroidectomy, dental avulsion) [74] or obstetric trauma [39]. In a cohort of 205 patients, Stone reported that 26% experienced an apparent “acceleration” of disease progression following stress or surgery; among these, 68% of women reported disease exacerbation during pregnancy or the postpartum period [24]. Other authors have described a history of craniofacial trauma and head injury [75,76], orbital and zygomatic fractures [75,77], dental procedures and dento-maxillary trauma [78,79], as well as facial surgical interventions [75,77] in patients with PRS. Wartenberg was among the first to propose, in 1945, the hypothesis that trauma might act as a triggering factor for PRS [80], a theory subsequently adopted by other authors. According to this hypothesis, trauma could initiate the atrophic process either through direct tissue injury [81,82] or by exposing tissue antigens that subsequently trigger an autoimmune response [83]. At present, trauma is rarely accepted as a direct causal factor and is more often regarded as a possible coincidental event rather than a definitive etiological determinant of PRS [84].

In some cases, an infectious etiology has been considered, with certain viral or bacterial agents proposed as potential triggers of PRS [85], although no specific pathogen has been isolated from cerebral tissue. Suspected agents include neurotropic viruses (e.g., herpes viruses) and *Borrelia burgdorferi* (Lyme disease) [86]. Additionally, infections such as otitis, dental infections, rubella, diphtheria, syphilis, and tuberculosis have been reported in the medical history of patients with PRS [39,87,88]. Although causality remains uncertain [12,29], some authors continue to support the hypothesis of a viral infection acting as a triggering factor [81,82]. The main mechanisms proposed to explain the infectious theory include direct infection and postinfectious autoimmunity. On the one hand, neurotropic viruses may infect and damage facial tissues or cranial nerves directly [83]; on the other hand, infection may induce an autoimmune response through molecular mimicry and antigen release. Nevertheless, direct microbiological evidence of infection in PRS is rare and inconsistent, and, as in the case of our patient, many individuals with PRS report no history of preceding infection.

The hypothesis of cervical sympathetic trunk dysfunction is supported by experimental animal studies demonstrating clinical features resembling PRS following ablation of the superior cervical ganglion. In these models, animals developed hemifacial, osseous, and muscular atrophy, as well as ipsilateral enophthalmos on the side of sympathectomy [72,74,88,89]. In some patients with PRS, signs of autonomic dysfunction have been reported, including ipsilateral Horner syndrome [51,90]. However, other authors have documented normal responses on autonomic function testing [74]. Moreover, in certain cases, sympathectomy appeared to halt disease progression, supporting the hypothesis that sympathetic irritation may contribute to the development of facial hemiatrophy [2]. Hyperactivity of brainstem centers has also been proposed as a potential pathogenic mechanism underlying PRS [47].

### 3.3. Pathological Findings

Histopathological studies performed on biopsied atrophic skin tissue in PRS most commonly demonstrate epidermal atrophy [40], dermal sclerosis [40] with dense, thickened [12] and hyalinized collagen bundles [41], as well as loss of the normal dermal architecture [40]. Perivascular lymphocytic inflammatory infiltrates have also been described [2,12,91,92], along with atrophy of skin appendages, reduced hair follicles, and atrophy of sweat glands [40]. The subcutaneous tissue has been shown to exhibit marked atrophy and significant volume loss, often accompanied by subcutaneous fibrosis [50]. From a vascular perspective, thickening of the vascular walls with partial luminal obliteration due to intimal proliferation has been identified [50,91]. Additionally, degenerative cortical changes [85,93], perivascular lymphocytic infiltrates with pial and glial proliferation in the white and gray matter of the hemisphere ipsilateral to the facial atrophy [80], microvascular malformations [85,93] and leptomeningeal fibrosis [85,93] have also been reported.

### 3.4. Clinical Manifestations

In PRS, facial manifestations are cardinal, with the defining feature being progressive unilateral hemifacial atrophy involving the skin, subcutaneous tissue, muscles, and, in some cases, the underlying bone structures [2]. Clinically, the condition presents as progressive facial asymmetry within the distribution territory of the trigeminal nerve. Characteristic features include a “sunken” hemiface, deviation of the nose and/or mouth toward the affected side, and enophthalmos secondary to loss of orbital fat [2].

Over time, several authors have attempted to develop classification systems for PRS based on the severity of soft-tissue atrophy and the extent of facial bone involvement. Among the most widely recognized are the system proposed by Guerrerosantos et al. in 2007 [23], the classification introduced by Hu et al. in 2011 [94], and the Raposo-do-Amaral system published in 2014 [95]. However, their adoption has not been universal, and many centers continue to rely on institutional protocols or ad hoc descriptive assessments of disease severity [96,97]. The lack of multicenter validation and comparative studies further limits the global acceptance of any single classification system [95].

As the classification system proposed by Guerrerosantos et al. (2007) has remained influential in the reconstructive surgical literature due to its clinical illustrations and case series [23], we elected to apply this system in the present case report. This classification stratifies PRS into four clinical subtypes of increasing severity, based on the clinician’s subjective assessment of tissue depression: depression perceptible only to the patient (type 1), depression involving the skin or cartilage (type 2), osseous involvement (type 3), and cutaneous tissue directly overlying bone (type 4) [23].

Although PRS is primarily a clinical diagnosis, imaging plays a crucial role in assessing the extent of cutaneous, muscular, and osseous atrophy, detecting intracranial involvement, and monitoring disease progression [98,99]. In addition to the well-recognized local cutaneous, ophthalmologic, and maxillofacial manifestations, PRS may also be associated with systemic features, including neurological, endocrinological, immunological, cardiac, and infectious involvement [2].

#### 3.4.1. Cutaneous Manifestations

Early dermatological signs are often subtle and may precede overt facial atrophy by months or even years. Initial cutaneous changes include hyperpigmentation or hypopigmentation [100,101] in the periorbital region, on the cheek, or along the mandibular area, sometimes preceded by trigeminal neuralgia [102]. The skin gradually becomes thinned, wrinkled, and dry, acquiring a characteristic “parchment-like” appearance [2,102]. Alopecia areata may represent an early manifestation, occurring in the affected region prior to the development of clinically evident atrophy [2,16], as observed in our patient, whose PRS presented atypically with alopecia in the left parietal scalp region.

In some cases, patients present with linear areas of cutaneous sclerosis, known as the “en coup de sabre” lesion, which has been reported in approximately 25% of PRS cases [41] and was also observed in the present case. Conversely, up to 28% of patients with linear “en coup de sabre” lesions exhibit features overlapping with PRS, such as progressive hemifacial atrophy or similar histopathological findings on skin biopsy [51,103]. Moreover, progression from linear scleroderma to PRS has been documented, further complicating the differentiation between these two disorders [2,104]. This clinicopathological overlap has generated ongoing debate regarding the relationship between the two entities, with some authors proposing that they represent variants within the same disease spectrum [105,106]. However, in linear scleroderma, unlike PRS, deeper craniofacial structures are typically not involved [17,104].

Cutaneous changes progress gradually, leading to loss of subcutaneous fat and tissue retraction in the malar, periorbital, or mandibular regions [16,17]. This skin retraction may result in deviation of the mouth and nose toward the affected side [2,107], a finding also observed in our patient, in whom the left oral commissure was retracted and deviated ipsilaterally. The progressive atrophy ultimately leads to marked facial asymmetry, which becomes increasingly evident as the disease advances [15,108], eventually contributing to the establishment of the diagnosis of PRS. The most common cutaneous manifestations of PRS are summarized in Table 1.

#### 3.4.2. Neurological Manifestations

Neurological manifestations are reported in approximately 15–45% of patients with PRS [17,102,109,110], with some authors describing rates as high as 58% [2], leading to its classification as a neurocutaneous syndrome [17,102,109,110]. These neurological manifestations may occur concomitantly with, or even precede, the onset of facial atrophy [17,102].

The most frequently reported neurological symptoms in PRS include seizures [24,37,110,111,112,113,114,115,116], headache, facial pain, and migraine [24,69,117], which in some cases may be associated with hemiplegia [14,118]. Epilepsy has been reported in up to 45% of patients in certain series and may present as either focal or generalized seizures [102]. Migraine has been described in approximately 30% of patients with PRS [50], while facial pain has been reported in up to 46% of cases [56], including neuropathic pain such as trigeminal neuralgia [119,120]. Trigeminal nerve involvement may manifest as chronic facial pain and trigeminal neuralgia [25,117,121,122], and in vivo confocal microscopy studies of the cornea have demonstrated reduced trigeminal nerve fiber density on the affected side compared to the contralateral side [25]. In some patients, late-onset psychiatric manifestations such as anxiety, panic attacks, depression, agoraphobia, or, rarely, psychosis have been described [2,41]. Cognitive impairment may also occur, occasionally as the sole manifestation of hemispheric cerebral atrophy [123]. Fortunately, in our patient, PRS manifestations were limited to cutaneous and muscular involvement of the face, without associated neurological symptoms. The neurological manifestations reported in PRS are summarized in the table below.

#### 3.4.3. Ophthalmologic Manifestations

Ophthalmologic complications have been reported in approximately 10–35% of cases, the most frequent (ordered by severity) being optic nerve damage, retinitis, and uveitis, as well as retro-orbital fat atrophy leading to enophthalmos [2,110,116]. Among these, enophthalmos represents the most common ophthalmologic manifestation, resulting from loss of retrobulbar fat and atrophy of the orbital tissues [15,16,17]. This posterior displacement of the globe contributes to facial asymmetry and may be detected through neuroimaging [124]. In our patient, likely due to the relatively mild disease presentation, no ocular or orbital abnormalities were identified. Nevertheless, we consider a comprehensive ophthalmologic evaluation to be recommended in all patients with PRS in order to enable early detection and monitoring of potential ophthalmologic complications. The most common ocular manifestations associated with PRS are summarized in Table 2.

#### 3.4.4. Oral and Maxillofacial Manifestations

Dental and orofacial abnormalities are common in PRS and may provide important diagnostic clues. Reported manifestations include deviation of the dental midline, atrophic dental roots, root resorption [125,126], root exposure, delayed tooth eruption [125], dilaceration [125], and gingival involvement [2,41,102,127,128]. Teeth on the affected side may appear smaller than those on the contralateral side [17], and certain anomalies, such as short roots, may occur and remain underdiagnosed [104].

Atrophy may also involve the lips and tongue, leading to absence of lingual papillae on the affected side and atrophy of the upper lip [15,128]. Progressive maxillary and mandibular hypoplasia may result in dental malalignment and masticatory difficulties [16,127]. Deviation of the nose and lips toward the affected side represents a frequent clinical feature [15]. Although soft-tissue atrophy involving the skin, subcutaneous fat, and muscles constitutes a hallmark sign of PRS, osseous involvement has been reported only in a subset of cases [17,29,129]. Documented skeletal changes include atrophy of the zygoma, maxilla, ethmoid bone, orbit, and mandible, typically evaluated using radiography and CT imaging [17,29,129,130].

With regard to etiopathogenesis, several theories have been proposed over the years, including atrophy secondary to cutaneous retraction [131], atrophy resulting from muscular retraction [132], and primary osseous atrophy due to bone ischemia caused by small-vessel vasculitis [133]. Moreover, vasculitis, also observed in scleroderma, may affect branches of the internal maxillary artery supplying the mandibular angle, mandibular condyle, and coronoid process, three anatomical regions that are predominantly involved in PRS [130,133]. In terms of maxillofacial tumoral manifestations associated with PRS, isolated cases of mandibular odontogenic fibroma [68], odontogenic cysts [134], and mandibular odontoma have been reported [135].

#### 3.4.5. Endocrine and Autoimmune Manifestations

Thyroid disorders represent the most frequently reported category of endocrine manifestations in PRS, although their exact prevalence remains unknown. The literature documents associations with hypothyroidism [83,136,137], hyperthyroidism [83], and autoimmune thyroid diseases such as Hashimoto thyroiditis and Graves’ disease [2,63,83]. Several authors have also described associations between PRS and acromegaly secondary to a growth hormone-secreting pituitary adenoma [63], multiple endocrine neoplasia type 1 with pituitary adenoma [138], and diabetes mellitus [82]. Other reported endocrine abnormalities include lipodystrophies [63,83], primary disorders of calcium metabolism [139], and disturbances in lipid metabolism [81,82,140]. Although these observations suggest possible associations, a causal relationship has not been clearly established.

With regard to autoimmune manifestations, PRS has been associated with multiple autoimmune disorders [40]. It is frequently considered part of the morphea spectrum, given the histopathological overlap observed between the two conditions [26,29]. The main endocrine and autoimmune manifestations reported in PRS are summarized in Table 3.

### 3.5. Imaging Findings

Imaging is essential for evaluating the extent of involvement in PRS, as approximately 20–50% of patients demonstrate abnormal radiological findings, which are typically ipsilateral to the affected side of the face [99,100]. Moreover, certain intracranial abnormalities may remain subclinical [17,111,141]. Current recommendations suggest that all patients presenting with cutaneous manifestations limited to the face or scalp, regardless of the presence or absence of neurological symptoms, should undergo brain MRI at the time of diagnosis [142].

MRI examinations in patients with PRS most commonly demonstrate craniofacial soft-tissue atrophy, including calvarial thinning [17,34], findings that were also identified in the present case. In our patient, MRI revealed ipsilateral atrophy of the skin, subcutaneous tissue, and temporalis and masseter muscles, as well as focal thinning of the left frontal bone. At the facial level, MRI typically shows loss of soft-tissue volume with asymmetric atrophy of the subcutaneous fat and facial musculature [102,124], as well as enophthalmos, which can be demonstrated on coronal T2-weighted and axial T1-weighted images [124].

Imaging studies have also identified parotid gland abnormalities in patients with PRS, including parotid gland volume loss and increased fat content, reflected by elevated T1- and T2-weighted signal intensity, most commonly ipsilateral to the facial atrophy [34]. Gorolay et al. reported parotid gland abnormalities in 38% of 24 patients with PRS, with parotid gland volume loss being the most frequent finding [34]. In our case, MRI demonstrated volume reduction in both the parotid and submandibular salivary glands on the same side as the facial atrophy, consistent with previously reported imaging findings of salivary gland involvement in PRS.

Intracranial abnormalities are most commonly ipsilateral to the side of facial involvement [73,92,110,119,143], supporting theories that directly link neurological manifestations to the cutaneous disease process [144]. The most frequent MRI finding is the presence of white matter hyperintensities on T2-weighted and FLAIR sequences, typically located in the deep white matter ipsilateral to the facial atrophy [17,141,145]. These changes are most commonly observed in the frontal lobe [13], as in the present case. Importantly, the lesions were not periventricular in distribution, a pattern more commonly associated with primary demyelinating or degenerative processes. Morphologically, these lesions are small and non-enhancing, without mass effect, features that are more consistent with chronic microangiopathic changes. Accordingly, they are considered to reflect long-standing tissue injury secondary to low-grade ischemic mechanisms or chronic neuroinflammatory processes [92].

Ipsilateral cerebral atrophy represents another frequently reported finding and may involve the cerebral cortex, white matter, and deep brain structures [17,123,141]. The atrophy may be subtle, presenting as mild cortical sulcal effacement and thinning of the overlying scalp soft tissues [16,108]. In more severe cases, ipsilateral ventriculomegaly may also be observed [17]. In our patient, no imaging signs of cerebral atrophy were identified.

Some cases of PRS demonstrate mild cortical thickening and leptomeningeal enhancement following contrast administration, suggesting an active inflammatory process [92,145]. These changes may also be associated with dense mineral deposits [92].

Advanced MRI techniques provide additional insights into cerebral pathology in PRS. Susceptibility-weighted imaging (SWI) can detect punctate microhemorrhages and calcifications that may not be visible on conventional sequences [17]. Magnetic resonance spectroscopy (MRS) studies have demonstrated decreased N-acetylaspartate (a marker of neuronal integrity), increased choline and myo-inositol levels (markers of inflammation and gliosis), and the presence of a prominent lipid peak at 0.8–0.9 ppm within affected regions [141,145]. Diffusion tensor imaging (DTI) may reveal reduced fractional anisotropy in the involved white matter, indicating disruption of microstructural integrity [141,145]. Additionally, apparent diffusion coefficient (ADC) values are increased in affected regions compared with the contralateral side [145]. Perfusion imaging may demonstrate reduced cerebral perfusion in involved areas [145], while most metabolic neuroimaging studies report a relative decrease in the choline-to-creatine (Cho/Cr) ratio and reduced N-acetylaspartate levels [13,31,146,147].

Craniofacial CT provides complementary information to MRI and is particularly useful for evaluating osseous changes and intracranial calcifications [17,148]. CT clearly demonstrates facial skeletal asymmetry, including hypoplasia and resorption of the maxilla, mandible, or zygomatic arch [17,127,148]. These findings are essential for reconstructive surgical planning [148]. CT may also reveal abnormalities of the nasal cavity and paranasal sinuses, including maxillary sinus hypoplasia [106]. Furthermore, CT is superior to MRI in detecting parenchymal calcifications, which may be present in the frontal or occipital lobes or within the basal ganglia, typically ipsilateral to the facial atrophy [16,17]. These calcifications may appear discrete or linear and are considered markers of long-standing disease [17]. CT can also demonstrate thinning of the skin and subcutaneous tissues; however, MRI is generally more sensitive for assessing these soft-tissue changes [17,149].

Other complementary imaging modalities include Doppler ultrasonography of the head and neck vessels and nailfold capillaroscopy, which may be used to assess vascular supply and detect vascular sclerosis [102]. However, ultrasonography is not recommended as a primary diagnostic modality for PRS, as most studies rely on MRI and CT for detailed anatomical and cerebral characterization [16,102]. Angiographic studies are usually normal in PRS, which may assist in differentiating the condition from other cerebral vasculopathies [92]. Nevertheless, some reports have described narrowed vascular lumens detected on ultrasound examination [141]. The most common imaging findings in PRS are summarized in the table below (Table 4):

Although no universally accepted imaging staging system exists, some centers have proposed classifications based on the degree of subcutaneous atrophy and dermal changes observed on MRI [16,149]. Schlecht et al. introduced an MRI-based staging system comprising several stages: stage 1—thinning of the subcutaneous tissue; stage 2—loss of dermal-subcutaneous interdigitation with a “smooth” dermal appearance; and stage 3—complete absence of a clearly demarcated subcutis [149]. Studies have demonstrated a tendency toward concordance between MRI-based staging and clinical staging [149]. However, these staging systems are not internationally standardized diagnostic criteria and are primarily used for monitoring disease progression and treatment response.

### 3.6. Differential Diagnosis

The diagnosis of PRS remains a clinical challenge due to the rarity of the condition, variability in presentation, and the absence of standardized diagnostic criteria. A comprehensive diagnostic approach is required, integrating detailed clinical evaluation with advanced imaging studies and, in selected cases, laboratory testing and histopathological examination. The differential diagnosis of PRS includes multiple conditions that may present with facial atrophy or asymmetry, necessitating careful clinical and radiological assessment to ensure accurate differentiation [16,17].

#### 3.6.1. Linear Scleroderma “En Coup de Sabre”

Linear scleroderma “en coup de sabre” represents the most important entity in the differential diagnosis of PRS, given the significant clinical, histological, and imaging overlap between the two conditions [16,17,105,106]. Some authors consider PRS and linear scleroderma to be variants within the same disease spectrum, both representing forms of localized scleroderma [15,105,106]. Linear scleroderma is characterized by linear frontoparietal cutaneous sclerosis, which may be accompanied by deeper tissue atrophy [16,17]. In contrast to PRS, where cutaneous sclerosis is minimal or absent, linear scleroderma typically exhibits more pronounced skin induration [102]. Histopathological examination may aid in differentiation: PRS is characterized by atrophy with variable chronic perivascular inflammation and relatively preserved dermal elastic tissue, whereas linear scleroderma demonstrates more prominent dermal sclerosis [124]. On imaging, both PRS and linear scleroderma may present with ipsilateral cerebral lesions, including white matter hyperintensities, cerebral atrophy, and calcifications [34,105,106]. Distinguishing between the two entities requires thorough clinical assessment, imaging evaluation, and, in some cases, skin biopsy [16,17].

#### 3.6.2. Hemifacial Microsomia and Goldenhar Syndrome

Hemifacial microsomia and Goldenhar syndrome are congenital malformations present at birth, characterized by hypoplasia of structures derived from the first and second branchial arches [15,16,17]. In contrast to PRS, which is an acquired and progressive condition, these syndromes are non-progressive and manifest from birth [15,16,150]. Clinical features that aid in differentiation include the age at onset (congenital vs. acquired during childhood or adolescence), disease course (static vs. progressive), and cutaneous changes (typically absent in hemifacial microsomia but present in PRS) [15,16]. A detailed medical history and documentation of progression over time are essential for distinguishing these entities from PRS [16].

#### 3.6.3. Rasmussen Encephalitis

Rasmussen encephalitis is a rare neurological disorder characterized by chronic unilateral cerebral inflammation, progressive hemispheric atrophy, and refractory epilepsy [16,17]. Although it may produce unilateral cerebral atrophy and epilepsy similar to PRS, its clinical course and neuroimaging and immunological findings are distinctive [17]. Key differences include the predominance and severity of neurological manifestations in Rasmussen encephalitis, typically marked by intractable seizures and progressive neurological deficits, compared with the absence or mild nature of neurological symptoms in many cases of PRS. In addition, facial atrophy is absent or minimal in Rasmussen encephalitis, whereas it represents a defining feature of PRS [16,17]. From a neuroimaging perspective, cerebral atrophy in Rasmussen encephalitis is typically rapidly progressive, in contrast to PRS, in which changes are often more subtle [145]. Brain biopsies in some PRS cases have demonstrated perivascular lymphocytic infiltrates with vasculitic features, gliosis, and leptomeningeal sclerosis, findings that may mimic those observed in Rasmussen encephalitis, thereby underscoring the importance of careful clinicoradiological correlation [106].

#### 3.6.4. Other Differential Diagnoses

Barraquer–Simons syndrome (acquired partial lipodystrophy) represents another important differential diagnosis. It is characterized by progressive loss of adipose tissue, often with a bilateral distribution and, in some cases, a history of triggering events. Unlike PRS, facial involvement in Barraquer–Simons syndrome is typically bilateral and not associated with progressive cutaneous sclerosis or focal craniofacial atrophy confined to one side [15,16]. HIV-associated lipoatrophy may also result in facial fat loss; however, the clinical context, namely HIV infection and exposure to antiretroviral therapy, facilitates differentiation [102]. Sturge–Weber syndrome should also be considered, as it may present with hemispheric cerebral atrophy. However, it is typically associated with a characteristic facial capillary malformation (port-wine stain) and gyriform intracranial calcifications [145]. Other differential diagnoses include deep tissue atrophy secondary to corticosteroid injections [102], as well as syringomyelia or cranial nerve tumors. Although these conditions may produce facial asymmetry, they exhibit distinctive imaging characteristics that allow differentiation from PRS [102].

### 3.7. Literature-Based Diagnostic Framework for Parry–Romberg Syndrome

#### 3.7.1. Methodology for the Development of the Proposed Diagnostic Framework

The proposed diagnostic framework for PRS was developed based on a structured synthesis of data from the medical literature and the clinical-imaging characteristics observed in the present case. Given the absence of universally accepted diagnostic criteria for PRS, it was not possible to perform a systematic meta-analytic review. Instead, a comprehensive narrative review of the literature was conducted, focusing on case reports, case series, and review articles describing the clinical, neurological, and imaging spectrum of the disease.

The main clinical features, imaging findings, and associated systemic manifestations were extracted and grouped according to their frequency of reporting and consistency across independent studies. Particular attention was given to elements repeatedly described in association with PRS, including progressive hemifacial atrophy, unilateral distribution, the typical clinical course, and intracranial abnormalities identified on imaging.

It is important to emphasize that this framework does not represent a validated scoring system or standardized diagnostic criteria but rather a practical, literature-based synthesis intended to support clinical reasoning and to contribute to a more standardized approach in the future evaluation of this rare disease.

#### 3.7.2. Proposed Literature-Based Clinical Diagnostic Framework for Parry–Romberg Syndrome

The current literature does not provide universally accepted and internationally validated diagnostic criteria for PRS [16,17,34,141]. Most studies consider the diagnosis to be primarily clinical, supported by imaging findings and, when necessary, histopathological examination and autoimmune testing [16,17,141]. Chiu et al. define PRS simply as “hemifacial atrophy,” emphasizing the predominantly clinical nature of the diagnosis [106]. Although imaging-based staging systems and practical criteria have been proposed by individual centers, these remain inconsistently adopted and do not constitute an international standard [149]. The absence of standardized criteria reflects the rarity of the disease, the variability of its clinical presentation, and the incomplete understanding of its pathogenesis [16,127]. Despite the lack of formal criteria, the literature converges on several essential elements for the diagnosis of PRS.

Based on these key features, we propose a practical, literature-based clinical framework intended to assist clinicians in the diagnostic evaluation of suspected PRS, summarized in Table 5. It is important to emphasize that this framework does not represent validated diagnostic criteria and should not be interpreted as mandatory or exclusionary. Rather, it should be used as a supportive tool within a comprehensive clinical assessment.

Based on current evidence, we propose a practical diagnostic algorithm for the assessment of patients with suspected PRS (Table 6).

### 3.8. Treatment

The management of PRS remains challenging, as no standardized treatment guidelines are currently available due to the rarity of the condition. The primary therapeutic goals are to slow disease progression and alleviate associated symptoms, although treatment response varies considerably among patients [1,17]. In cases with intracranial involvement, immunosuppressive therapy may be considered [12,14,26,151]. Local treatment options include topical corticosteroids [26], botulinum toxin injections [26], vitamin D analogs [12], including psoralen plus ultraviolet A (PUVA) therapy [26], emollients [12], and phototherapy [12,152]. Systemic therapeutic options that have been reported include corticosteroids [12,26,151], methotrexate [12,26,151], often in combination with oral corticosteroids [26], procaine penicillin [19], D-penicillamine [26], hydroxychloroquine [12,26], tetracycline [26], cyclosporine [12], vitamin E [26], and retinoids [12].

With regard to surgical management, reconstructive procedures are generally recommended after disease stabilization, typically following a monitoring period of 1–2 years to ensure the absence of active progression. Surgical treatment aims at facial reconstruction through augmentation of the atrophic regions and restoration of facial symmetry [12,31,153]. Therapeutic strategies consist of a combination of facial contour remodeling and volumetric restoration [23,153,154,155,156,157,158,159,160,161,162,163,164,165,166,167,168,169,170,171,172], which may be performed either as a single surgical procedure (Table 7) or through multiple combined surgical interventions (Table 8), depending on the severity of facial atrophy.

Additionally, patients should receive psychological support, given the emotional and psychosocial implications of PRS [16]. Regardless of the therapeutic option selected, these treatment modalities provide aesthetic improvement only temporarily, as outcomes tend to diminish over time due to gravitational effects, often necessitating further surgical intervention [140].

## 4. Conclusions

PRS is a rare neurocutaneous disorder characterized by progressive unilateral hemifacial atrophy, with potential intracranial involvement, even in the absence of overt neurological manifestations. The present case highlights a moderate form of the disease (stage 2 according to the Guerrerosantos classification), associated with ipsilateral subcortical white matter lesions on MRI, without significant clinical neurological impairment. This radiological–clinical discrepancy underscores the potentially subclinical nature of central nervous system involvement in PRS. Prospective multicenter studies are warranted to validate standardized diagnostic and staging criteria correlated with clinical and imaging findings, as well as to further elucidate the pathogenic mechanisms and identify reliable biomarkers of disease activity. Standardization of imaging protocols and evaluation of the efficacy of immunomodulatory therapies may contribute to optimizing the management of this rare condition.

## Figures and Tables

**Table 1:** Cutaneous and neurological manifestations in Parry–Romberg Syndrome.

Cutaneous Manifestations	Neurological Manifestations	
Band-like alopecia Linear scleroderma “en coup de sabre” Morphea Hyperpigmentation Port-wine stain Lupus Ipsilateral vitiligo Raynaud phenomenon Klippel–Trénaunay syndrome Hemangiomas Poliosis	**Craniofacial pain**	**Cerebellar manifestations**
	Facial pain Headache Migraine	Cerebellar syndrome Nystagmus Gait disturbance
	**Motor disturbances**	**Cranial nerve involvement**
	Hemiplegia Limb weakness Limb atrophy Trunk atrophy Paroxysmal kinesigenic dyskinesia Piramidal signs Mirror movements/synkinesia	Trigeminal neuralgia Pseudoptosis (isolated ptosis) Vision loss Diplopia Oculomotor nerve palsy Tinnitus Facial nerve palsy Homonymous hemianopsia Deafness Anisocoria (miosis or mydriasis on the affected side)
	**Sensory disturbances**	**Neuropsychiatric disorders**
	Dysesthesia Hypesthesia Hemianesthesia	Mental retardation Hallucinations Intellectual disability Psychiatric disorders
	**Central neurological symptoms**	**Digestive troubles**
	Seizure Amnesic aphasia Unilateral alien hand syndrome	Dysphagia Gastroparesis
	**Spasms**	**PNS disorders**
	Facial spasms Masticatory spasms Limb spasm	Polyneuropathy Decreased deep tendon reflexes

**Table 2:** Ocular manifestation in Parry–Romberg syndrome.

Conjunctiva	Cornea	Sclera
Palpebral pigmentation	Keratopathy Reduced corneal nerve density Flour-like stromal deposits Reduced corneal sensation Refractive changes Photophobia Corneal precipitates	Spontaneous scleral melting Episcleritis
		**Vitreous**
		Vitritis
**Uveal tract**	**Retina**	**Miscellaneous**
Anterior uveitis Iris atrophy Iris crystalline deposits Fuchs Iridocyclitis Panuveitis Uveitis Cyclitis Ciliary body hypotony	Retinal vasculitis Telangiectasia Pigmental epithelial changes Edema Retinitis pigmentosa Retinal detachment Coats disease Chorioretinal atrophy Occlusion of central retinal artery Retinal folding	Glaucoma Cataract Increase in pre-existing hyperopia

**Table 3:** Endocrine and autoimmune manifestations in Parry–Romberg syndrome.

Endocrine Manifestations	Autoimmune Manifestations
Hyperthyroidism Hypothyroidism Hashimoto thyroiditis Graves’ disease	Scleroderma Systemic lupus erythematosus Rheumatoid arthritis Ankylosing spondylitis Inflammatory bowel disease Primary biliary cholangitis Multiple sclerosis Sjögren disease Autoimmune hemolytic anemia

**Table 4:** Imaging findings on CT and MRI in Parry–Romberg syndrome.

Intracranial lesions	Intracranial vascular lesions
Brain atrophy Ipsilateral porencephaly Loss of cortical gyration Gyral enhancement Brain calcifications Mild cortical thickening Ipsilateral white matter hyperintensities (frontal, parietal, temporal, occipital lobes; subcortical regions; brainstem; optic nerve) Gray matter hyperintensity Cystic leukoencephalopathy Agenesis of head of the caudate nucleus Ipsilateral basal ganglia lesions	Intracranial aneurysm Vascular malformations Cerebral microhemorrhages Unilateral focal corpus callosum infarctions Ipsilateral infarcts in amygdaloid body Acute ischemic or hemorrhagic stroke Hypoplasic supraoptic arteries Stenosis or occlusion of the supraoptic arteries Cerebral cavernous malformation
**Skull and meningeal lesions**	**Miscellaneous**
Dense mineral deposition or calcification Dural mater atrophy Leptomeningeal enhancement Ipsilateral meningeal lesions	Subdural hygroma Syringomyelia Ventriculomegaly Isolated asymmetric ventricles Non-vascular brain tumors Orbital muscle thickening

**Table 5:** Literature-based clinical and supportive features of Parry–Romberg syndrome.

Key Clinical Features	
Progressive hemifacial atrophy	Defining feature involving the skin, subcutaneous tissue, adipose tissue, muscles, and occasionally the underlying bone or brain parenchyma [16,17,148]
Onset in childhood or adolescence	Typical onset within the first two decades of life [16,17,128]
Unilateral distribution	Usually confined to one side of the face, without crossing the midline [15,16]
Slow and progressive course	Progressive course over 2–20 years, followed by stabilization [15,16]
Absence of congenital craniofacial anomalies	Exclusion of congenital craniofacial malformations [15,16]
**Supportive Investigational Findings**	
Brain and neck MRI	MRI evidence of ipsilateral atrophy, T2/FLAIR white matter hyperintensities, or other intracranial lesions [17,141,148]
Craniofacial CT scan	CT demonstration of osseous changes (e.g., maxillary or mandibular hypoplasia) and intracranial calcifications [17,127,148]
Skin biopsy (optional)	Histopathological findings compatible with morphea/localized scleroderma or exclusion of other dermatoses [16,17]
Autoimmune laboratory testing (optional)	Detection of autoimmune markers supporting a possible autoimmune etiology [17,123,124]

**Table 6:** Proposed practical diagnostic algorithm for Parry–Romberg syndrome.

Step 1. Initial clinical evaluation	
Comprehensive medical history	Age at symptom onset, duration and progression of disease, and prior history of trauma or infection
Complete physical examination	Evaluation of the severity of facial atrophy, documentation of cutaneous changes, and comprehensive ophthalmologic and dental assessment
Focused neurological assessment	Screening for headache, facial pain, seizures, and focal neurological deficits
**Step 2. Imaging evaluation**	
Head MRI (mandatory)	Even in asymptomatic patients, to identify subclinical intracranial involvement [17,141,148]
Standard MRI sequences	T1-weighted, T2-weighted, FLAIR, and contrast-enhanced T1-weighted sequences
Advanced MRI sequences (optional)	SWI, magnetic resonance spectroscopy (MRS), diffusion tensor imaging (DTI), perfusion imaging [141,145]
Craniofacial CT scan	For assessment of osseous changes and intracranial calcifications, particularly when reconstructive surgery is being considered [17,127,148]
**Step 3. Laboratory testing**	
Routine laboratory tests	Complete blood count, erythrocyte sedimentation rate, C-reactive protein [123,124]
Autoimmune testing	ANA, anti-Scl-70, anti-dsDNA antibodies (particularly if morphea or systemic sclerosis is suspected) [17,123,124]
Serologic testing	To exclude infectious etiologies (e.g., Lyme disease, HIV, syphilis) [102]
Cerebrospinal fluid analysis (optional)	In cases with significant neurological manifestations [128,141]
**Step 4. Skin biopsy (selective)**	
Indications: - Atypical cutaneous features or rapid progression [16] - Consideration of immunosuppressive therapy with the need to confirm inflammatory activity [17] - Uncertain differential diagnosis with morphea or other dermatoses [17,102]	
**Step 5. Neurophysiological investigations (selective)**	
EEG	For patients with seizures or suspected epileptiform activity [102]
Evoked potentials, EMG, autonomic testing	In cases with complex neurological manifestations to assess the extent of involvement [141]
**Step 6. Multidisciplinary evaluation**	
Dermatology consultation for assessment of cutaneous changes and differentiation from morphea Neurology consultation for management of neurological manifestations Ophthalmology consultation for evaluation of ocular complications Maxillofacial surgery consultation for assessment of dental abnormalities and reconstructive planning	
**Step 7. Monitoring and reassessment**	
Periodic clinical follow-up to evaluate disease progression Repeat MRI at regular intervals (e.g., annually) during the active phase of the disease Reassessment of the need for immunomodulatory therapy or reconstructive surgery	

**Table 7:** Single-stage reconstructive surgical procedures in Parry–Romberg syndrome.

Facial Reconstruction	Flaps and Grafts	Volume Regeneration
Lipofilling Medpor^®^ implant (porous polyethylene implant) Porous polyethylene implant Cell-assisted lipotransfer	Tissue transfer using a free thigh fascioadipose flap Pedicled superficial temporal fascia adipofascial flap Perforator-based anterolateral thigh flaps Free vascular parascapular graft Vascularized serratus anterior muscle flap Thoracodorsal flaps Composite galea-frontalis flap Free groin flaps	Autologous fat transplantation Expanded polytetrafluoroethylene implant Poly-L-lactic acid

**Table 8:** Principal combined surgical procedures in Parry–Romberg syndrome.

Revascularized free flap * and dermis grafting Revascularized free flap * and lipoinjection Revascularized free flap * and Medpor^®^ (porous polyethylene) implant placement Revascularized free flap * and genioplasty Revascularized free flap * and revision liposuction
Coleman lipoinjection combined with transfer of a deepithelialized free parascapular flap Coleman lipoinjection and platelet-rich plasma gel Coleman lipoinjection and polyglactin-based material Lipoinjection combined with galeal flaps, free dermis-fat grafts, and osseous and cartilaginous grafts
Poly-L-lactic acid injection combined with lipofilling and intense pulsed light therapy Superficial temporal fascial flap and lipofilling

* Anterolateral thigh adipofascial flaps or latissimus dorsi flap.

## Data Availability

All data are reported in the text.
